# The Neutrophil: The Underdog That Packs a Punch in the Fight against Cancer

**DOI:** 10.3390/ijms21217820

**Published:** 2020-10-22

**Authors:** Natasha Ustyanovska Avtenyuk, Nienke Visser, Edwin Bremer, Valerie R. Wiersma

**Affiliations:** Department of Hematology, Cancer Research Center Groningen, University Medical Center Groningen (UMCG), University of Groningen, Hanzeplein 1/DA13, 9713 GZ Groningen, The Netherlands; n.ustyanovska.avtenyuk@umcg.nl (N.U.A.); n.visser@umcg.nl (N.V.)

**Keywords:** neutrophils, granulocytes, immunotherapy, cancer, phagocytosis, cytotoxicity

## Abstract

The advent of immunotherapy has had a major impact on the outcome and overall survival in many types of cancer. Current immunotherapeutic strategies typically aim to (re)activate anticancer T cell immunity, although the targeting of macrophage-mediated anticancer innate immunity has also emerged in recent years. Neutrophils, although comprising ≈ 60% of all white blood cells in the circulation, are still largely overlooked in this respect. Nevertheless, neutrophils have evident anticancer activity and can induce phagocytosis, trogocytosis, as well as the direct cytotoxic elimination of cancer cells. Furthermore, therapeutic tumor-targeting monoclonal antibodies trigger anticancer immune responses through all innate Fc-receptor expressing cells, including neutrophils. Indeed, the depletion of neutrophils strongly reduced the efficacy of monoclonal antibody treatment and increased tumor progression in various preclinical studies. In addition, the infusion of neutrophils in murine cancer models reduced tumor progression. However, evidence on the anticancer effects of neutrophils is fragmentary and mostly obtained in in vitro assays or murine models with reports on anticancer neutrophil activity in humans lagging behind. In this review, we aim to give an overview of the available knowledge of anticancer activity by neutrophils. Furthermore, we will describe strategies being explored for the therapeutic activation of anticancer neutrophil activity.

## 1. Introduction

In recent years, the implementation of cancer immunotherapy has yielded unprecedented clinical responses in many types of cancer. Most of the strategies pursued, e.g., using so-called checkpoint inhibitors [1] or chimeric antigen receptor T cells (reviewed in [2]), are aimed at restoring T cell anticancer immunity. More recently, strategies to (re)activate innate immunity by the targeted (re)activation of macrophages, such as CD47 blocking antibodies [3,4], have also entered clinical trials and have yielded promising early clinical responses [5]. A cell type that has so far been mostly overlooked as a potential source of anticancer immune activity is the neutrophil. The neutrophil is the predominant class of polymorphonuclear cell (PMN)/granulocyte (comprising ≈ 95% of total PMNs) and the most abundant cell type in the human bloodstream. Therefore, “neutrophil” will be used to describe all PMN-based studies in this review.

Neutrophils are widely recognized as the first line of defense in infectious disease, but they also have a clear modulatory role in cancer depending on the context and cancer stage [6,7]. Neutrophils at early stages of tumorigenesis are antitumoral [8], whereas a predominant protumoral role has been reported at established late stages of cancer [9]. In this respect, neutrophil infiltration associates with poor patient survival in various cancers [10,11,12,13]. On the one hand, tumor-associated neutrophils (TANs) have been associated with poor prognosis as has been described in for instance patients with diffuse large B-cell lymphoma (DLBCL) [11] and gastric cancer [14]. In this setting, the neutrophil–lymphocyte ratio (NLR) is also predictive for survival outcomes of cancer patients [15,16]. On the other hand patients with colorectal cancer had better overall survival rates when having high levels of TANs [17,18]. As for other tumor-infiltrated immune cells such as macrophages, polarization states with distinct pro- and antitumoral activity have been delineated for TANs that may account for this differential impact on cancer. 

The best known demarcation for neutrophils is that of N1 vs. N2 neutrophils, although by now, up to 19 different neutrophil subtypes have been reported (as reviewed by [19]). Neutrophils of the N2 subtype typically have tumor-promoting activity, with the key cytokine driving N2 differentiation being transforming growth factor beta (TGF-β) [20]. N1 neutrophils on the other hand are characterized by anticancer activity and an immunostimulatory expression profile, among others defined by high levels of tumor necrosis factor α (TNFα), Fas, intercellular adhesion molecule 1 (ICAM-1), and a low expression level of arginase [20,21]. The key cytokine driving N1 differentiation is interferon-beta (IFN-β) [22]. Interestingly, N1 neutrophils can also promote T cell immunity, with evident recruitment and activation of CD8^+^ T cells in tumor-bearing mice that was abrogated upon neutrophil depletion [23]. Differentation into a subtype is not a definitive state, with e.g., the blocking of TGF-β converting mature N2 TANs to an N1 phenotype in vivo [20]. A second way to classify neutrophil subsets is based on their density; i.e., low-density neutrophils (LDNs) or high-density neutrophils (HDNs). Neutrophils normally seggregate into the sedimentary fraction of leukocytes with segmented nuclei, leading to the term HDN. However, LDNs co-segregate with the mononuclear cell fraction during density-gradient isolation, and the fraction of LDN increases during (chronic) inflamation, e.g., as seen during auto-immunity and cancer [24]. Of note, HDNs are generally seen as antitumor, resembling N1 neutrophils, whereas LDNs are more immature and alike N2 neutrophils [24,25,26] 

Research on neutrophils in cancer is predominantly focused on the tumor-promoting role of N2 neutrophils in the tumor micro-environment (for an in-depth review, see [27]). Nevertheless, neutrophils can clearly have an antitumoral impact and promote anticancer immunity. For instance, such antitumor activity is evident from various murine models, with an injection of neutrophils in breast carcinoma-bearing rats increasing survival from 17% to 75% [28]. Reversely, the depletion of neutrophils in the same tumor model strongly decreased the rate of spontaneous regression from 87% to 30% [29]. Similarly, the depletion of neutrophils increased tumor relapse rates with 70% upon topical treatment of skin cancer-bearing mice with ingenol-3-angelate [30]. Importantly, the anticancer activity of monoclonal antibody treatment in murine models of melanoma and breast cancer was abrogated upon the depletion of neutrophils [31], with the re-infusion of neutrophils restoring anticancer activity. Human neutrophils also have clear antitumor activity, with neutrophils from healthy human donors displaying intrinsic anticancer activity toward cancer cell lines [32,33], which is an effect that is further increased by priming with granulocyte macrophage colony-stimulating factor (GM-CSF) [33]. 

For the elimination of cancer cells, neutrophils have a diverse set of cytotoxic tools at their disposal. Neutrophils can eliminate cancer cells by phagocytosis, which works most efficiently when the targeted cell is opsonized with (therapeutic) antibodies: a process called antibody-dependent cellular phagocytosis (ADCP). Since neutrophils are relatively small cells, they do not necessarily phagocytose complete target cells. Instead, they may take “bites” of the cancer cell membrane in a process termed trogocytosis. Trogocytosis leads to a loss of cell membrane integrity and, concomitantly, to cell death. Furthermore, neutrophils can trigger direct cytotoxicity by releasing high amounts of reactive oxygen species (ROS) or by the release of granule content, e.g., in the context of antibody-mediated targeting (also called antibody-dependent cellular cytotoxicity (ADCC)). Degranulation releases a host of cytotoxic molecules from primary, secondary, and tertiary granules that trigger the apoptotic elimination of cancer cells. 

**Figure 1:**
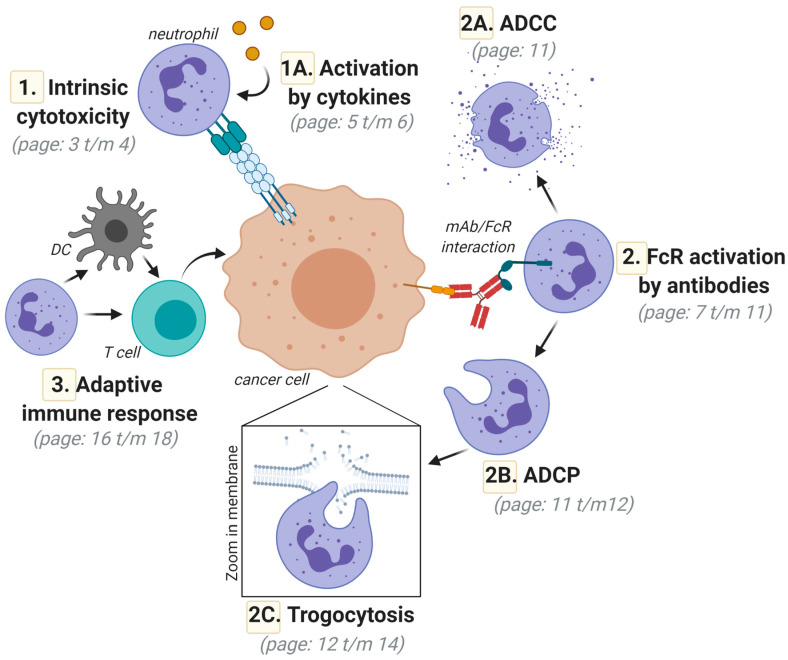
Overview of the review: the anticancer activity of neutrophils. **1.** Intrinsic cytotoxicity via death receptor signaling that is among others 1A. stimulated by cytokines. **2.** antibody-mediated Fc-receptor (FcR) activation includes: **2A.** Antibody-dependent cellular cytotoxicity (ADCC), **2B.** Antibody-dependent cellular phagocytosis (ADCP) and **2C.** Trogocytosis. **3.** Neutrophils trigger adaptive immune response by directly stimulating T cells or via the stimulation of antigen-presenting cells such as dendritic cells (DCs).

In this review, we will provide an in-depth overview of the antitumor activity of neutrophils and on neutrophil-mediated anticancer immunotherapy. Hereto, an overview of neutrophil biology and activation, including activation via antibody/Fc-receptor (FcR) interactions and cytokines, is given in the context of anticancer immune responses. Subsequently, the various mechanisms and therapeutic opportunities by which neutrophils can eliminate cancer cells will be detailed, focusing on ADCC, ADCP, and trogocytosis (Figure 1). 

## 2. Direct Neutrophil-Mediated Cytotoxic Activity toward Cancer Cells; Intrinsic Anticancer Activity via Death-Inducing Ligands of the Tumor Necrosis Factor (TNF) Superfamily

As described above, neutrophils have an intrinsic capacity to eliminate cancer cells, with various reports detailing the neutrophil-mediated elimination of cancer cell lines without affecting normal non-malignant epithelial or endothelial cells [32,33,34]. Furthermore, the injection of neutrophils isolated from healthy rats to breast carcinoma-bearing rats increased survival by 4-fold [28]. Interestingly, the amount of neutrophils in the circulation increased in tumor-bearing mice during tumor progression, with an increased cytotoxic activity of HDNs isolated from tumor-bearing compared to tumor-free animals [24]. Notably, neutrophils activated by pathogens, such as viruses and bacteria, can induce “collateral” damage toward cancer cells. The best known example hereof is the use of *Bacillus Calmette-Guérin* (BCG) immunotherapy for the treatment of bladder cancer, with neutrophils that respond to these bacteria also efficiently eliminating cancer cells [35]. In addition, the combination of polyinosinic–polycytidylic acid (polyI:C), a synthetic analog of double-stranded RNA that mimics viral infections, and inactivated viral particles suppressed melanoma tumor growth in a murine mouse model [23]. In both settings, the anticancer effect was abrogated upon the depletion of neutrophils. 

This intrinsic cytotoxic activity has been attributed to the expression of Tumor Necrosis Factor (TNF) superfamily ligands such as Fas ligand (FasL) [34] and tumor necrosis factor-related apoptosis-inducing ligand (TRAIL) on the surface of neutrophils [36,37,38]. In brief, the binding of membrane-expressed FasL or TRAIL can cross-link and activate the death receptors Fas and TRAIL-R1 and TRAIL-R2, respectively. Activation of these receptors triggers caspase-mediated apoptotic cell death. Indeed, neutrophil cytotoxicity toward lung cancer cells was dependent on FasL expression, as the blocking of Fas abrogated cytotoxicity [34]. Similarly, blocking either TRAIL or its receptors significantly inhibited the neutrophil-mediated cell death of leukemia cells [36,38]. In addition, neutrophils release the cleaved soluble form of TRAIL (sTRAIL), which (partly) retains cytotoxic activity. 

TRAIL can also be therapeutically used to augment neutrophil anticancer activity. For instance, we previously reported on an antibody fragment–TRAIL fusion protein that was designed to bind to C-type lectin-like molecule-1 (CLL-1) expressed on the surface of neutrophils [39]. The binding of this fusion protein, anti-CLL1:TRAIL, equipped neutrophils with high levels of surface TRAIL available for triggering TRAIL-R-mediated cell death in cancer cells. Indeed, TRAIL-mediated cytotoxic activity upon antiCLL-1:TRAIL treatment was strongly enhanced and also served to effectively potentiate ADCC induced by therapeutic antibodies on both solid and hematological cancers [39]. Of note, although neutrophils were reported to be sensitive to TRAIL-mediated cell death as well [37], [40], anti-CLL1:TRAIL did not negatively impact on neutrophil cell viability. Alternatively, TRAIL can be upregulated on the surface of neutrophils by stimulation using interferon-alpha (IFN-α) and interferon-gamma (IFN-γ) [36,37]. For instance, the ex vivo stimulation of neutrophils isolated from chronic myeloid leukemia (CML) patients with IFN-α led to the release of high levels of sTRAIL, and supernatants of these cultures induced apoptosis in leukemia cell lines [36]. In line with this finding, the serum levels of sTRAIL as well as leukocyte-associated membrane TRAIL were significantly increased in melanoma patients upon IFN-α treatment [37]. IFN-α not only positively regulates TRAIL expression but also sensitizes cancer cells to TRAIL-mediated apoptosis [41]. Correspondingly, circulating levels of sTRAIL have been shown to positively correlate with patient survival in various cancers, including renal cell and gall bladder carcinoma [42,43], although the contribution of neutrophils was not evaluated in these studies. IFN-α treatment also protects neutrophils from TRAIL-induced apoptosis [36]. Therefore, this cytokine may be of interest to stimulate surface and soluble levels of TRAIL without negatively affecting neutrophil viability. Of note, sTRAIL has been previously shown to only effectively activate one of its receptors, TRAIL-R1, whereas TRAIL-R2 signaling requires membrane TRAIL or oligomerized recombinant sTRAIL for the activation of pro-apoptotic signaling [44]. Thus, it will be interesting to dissect the role of sTRAIL in neutrophil-dependent anticancer activity in cancer types in relation to TRAILR1 and TRAILR2 expression. 

Thus, clinical evidence for the contribution of TRAIL in neutrophil-mediated cancer cell killing has been obtained from CML and melanoma patients, who are often treated with IFN-α. In addition, the anticancer effect as induced by BCG immunotherapy relies on the release of sTRAIL by neutrophils [45], with patients responding well to BCG immunotherapy having higher urinary levels of sTRAIL compared to non-responders [35]. Therefore, the intrinsic capacity of neutrophils to kill cancer cells at least partly relies on FasL and TRAIL expression. This can be exploited for anticancer therapy by arming neutrophils with additional TRAIL by either the use of neutrophil-targeting TRAIL fusion proteins or by stimulating TRAIL expression with cytokines such as IFN-α.

### Activation of Neutrophil-Mediated Anticancer Responses by Cytokines

The ability of neutrophil-activating cytokines to induce anticancer responses has been widely recognized for decades. For instance, GM-CSF primed neutrophils killed melanoma cell lines in co-culture experiments [33]. Similarly, murine melanoma cells that were genetically modified to secrete GM-CSF were strongly hampered in their in vivo growth [46]. These tumors were highly infiltrated by neutrophils, macrophages, and lymphocytes, which resulted in complete rejection in most cases. In line with this data, mice that did not express the beta-common chain (βc) receptor, a subunit essential for GM-CSF signaling, did not respond to vaccination with GM-CSF secreting melanoma cells [47]. Similar data were obtained for granulocyte colony-stimulating factor (G-CSF) [48], with increased ADCC by G-CSF primed neutrophils [46,49] and high neutrophil infiltration of cancer lesions in G-CSF secreting tumors [48]. Of note, lung cancer cells spontaneously secrete high levels of GM-CSF and G-CSF, which increased neutrophil longevity [50]. Further, expression levels in bronchoalveolar lavage fluid positively correlated with alveolar neutrophil counts [50]. The consequence of this for the cancer was not described, but it may be both tumoricidal as well as tumor promoting. In this respect, G-CSF and GM-CSF can indeed have protumoral functions and promote the differentiation of myeloid-derived suppressor cells, especially immunosuppressive neutrophils, from hematopoietic stem and progenitor cells [51,52], and they can facilitate cancer metastasis [53]. Importantly, these immunosuppressive effects were especially prominent during prolonged stimulation (3–5 days) with these cytokines [51], which argues for the implementation of short treatment with these cytokines during cancer therapy. 

In addition to the G-CSF and GM-CSF receptor, neutrophils express a multitude of cytokine receptors that can be categorized into three big families: conventional cytokine receptors (type I and type II), members of the interleukin 1 (IL-1)-receptors, and TNF-receptor superfamily members. All of these may aid in anticancer immune responses by neutrophils, with e.g., cancer cells engineered to secrete IL-2 triggering immediate neutrophil-mediated rejection of syngeneic tumors in mice [54,55,56]. Of note, IL-2 dose-dependently stimulates protein and RNA synthesis in GM-CSF primed neutrophils [57] and promotes adherence to endothelial cells [58], which may augment their activity. Furthermore, the expression of IL-4, IL-7, IL-10, IFN-α, and TNF-α in the same cancer model was, in all cases, associated with neutrophil as well as CD8^+^ T cell-mediated rejection [55]. Of these, TNF-α is of particular interest as it was identified as a key cytokine for the cytotoxic activity of neutrophils toward breast cancer cells [59] and increased neutrophil transmigration and nitric oxide release that promotes cancer cell killing [60]. In line with these data, serum levels of TNF-α in patients positively correlated with neutrophil cytotoxicity [59]. 

Another target for anticancer neutrophil activity is inhibition of the cytokine TGF-β, which is a multifunctional cytokine that is best known for its immune inhibitory roles in T cell biology. TGF-β also steers the phenotype of TANs toward an N2 pro-tumorigenic phenotype [20,61], inhibits neutrophil degranulation [62], and reduces the efficacy of G-CSF therapy [63]. This inhibitory effect of TGF-β on neutrophils argues for the use of TGF-β antagonists to improve neutrophil-mediated anticancer immune responses. Indeed, the blocking of TGF-β with neutralizing TGF-β monoclonal antibody (1D11) polarized TANs toward an N1 phenotype, yielding a significant increase in colorectal carcinoma cell death [64]. Furthermore, TGF-β blockade increased the level of neutrophil-attracting chemokines and increased the influx of N1 neutrophils into the tumor, with increased cytotoxic potential both in vitro and in vivo [20]. Of note, TGF-β can also have neutrophil-stimulating activity similar to N-Formylmethionyl-leucyl-phenylalanine (fMLP) [65]. Specifically, TGF-β has chemotaxis properties; it stimulates H_2_O_2_ and lactoferrin release in fibrogen-adherent PMNs, where the activation goes through the phospholipase D pathway [65]. These differential effects of TGF-β seem to depend on the cancer stage, as it suppresses early hepatocellular carcinoma development but has tumorigenic activity at later stages [66]. Thus, TGF-β inhibition in established late-stage malignancy is expected to promote neutrophil anticancer activity. Furthermore, TGF-β is also a crucial inhibitor of adaptive immunity, which may make TGF-β inhibition a double-edged sword that augments both innate and adaptive anticancer responses.

Interestingly, the blocking of TGF-β also restored the release of IFN-α/IFN-β in mice with mammary tumors, resulting in tumor rejection [67]. As described above, IFN-α and IFN-β drive the differentiation of TANs into an N1 antitumoral phenotype and trigger anticancer immunity [68,69]. Indeed, IFN-α has a pleiotropic positive impact on anticancer immunity and has been approved by the Food and Drug Administration (FDA) for the treatment of both solid and hematologic malignancies (reviewed by [70]), although the exact role of neutrophils in response to therapy remains to be determined. IFN-β has been shown to have direct cytotoxicity toward cancer cells in immune-incompetent mice [71], although it is also used to suppress autoimmune response in multiple sclerosis. Of note, the release of IFN-α as well as IFN-β can also be efficiently triggered by 5,6-dimethylxanthenone-4-acetic acid (DMXAA), which is a chemotherapeutic agent that causes cancer cell death by tumor vascular disruption and cytokine production. In this particular case, IFN-α and IFN-β are produced by macrophages [67,72]. Hence, increasing IFN-α/β levels may be used to simultaneously trigger direct tumoricidal as well as indirect neutrophil-mediated anticancer effects, which is further increased by blocking TGF-β [67]. 

In addition to cytokines, there are various chemokines that attract neutrophils to the site of infection or cancer. The best-known chemoattractant for neutrophils is IL-8, also known as CXCL8 or neutrophil-activating factor [73], which is also the main, or even only, chemokine that neutrophils can secrete themselves to create a positive feedback loop [74]. Although its role in anticancer immunity is still controversial, most reports claim a tumor-promoting role due to sustained neutrophil recruitment and inflammation, promoting metastasis [75] and reducing the the efficacy of checkpoint blockade [10,76]. Reversely, the chemokine receptor CXCR2 and its ligands CXCL1, CXCL2, and CXCL5 play an important role in homing of neutrophils to cancer cells to limit tumor growth. In this respect, the secretion of CXCL5 and IL-8 by renal cell carcinoma (RCC) cells recruited neutrophils and inhibited the formation of metastases [77]. In line with these data, only RCC with a low expression of neutrophil chemokines CXCL1, CXCL2, CXCL3, CXCL5, and IL-8 were able to metastasize. In addition, the receptors CCR2 and CCR5 were increased in pre-metastatic lung samples, which was probably due to expression on tumor-infiltrated neutrophils, and lung cancer cells were found to secrete their ligands CCL2 and CCL5 [63]. Indeed, in vitro treatment with CCL2 and CCL5 stimulated neutrophils to kill tumor cells [63]. In contrast, increased serum levels of chemokines CXCL1 and CXCL2 in mice correlated with increasing levels of CXCR2-expressing neutrophils in the blood and enhanced melanoma growth, with tumor growth being reduced by antibody-mediated blocking of CXCR2 [78].

Thus, the recruitment of neutrophils via cytokines and chemokines to cancer cells can have both anticancer as well as tumor-promoting effects, which are most likely depending on the type of neutrophils recruited and/or factors present in the micro-environment. Therefore, when designing cytokine-based or chemokine-based anticancer therapy with the goal of activating neutrophil-mediated anticancer immune responses, this should be taken into account. Firstly, local concentrations in the tumor microenvironment of the infused cytokine may heavily differ from systemic concentrations. Indeed, mice immunized with ex vivo GM-CSF-transfected tumor cells were better protected toward subsequent tumor challenge compared to mice immunized with parental tumor cells and the cutaneous transfection of GM-CSF cDNA at the vaccination site, which may be the result of four times higher local concentrations of GM-CSF in the tumor microenvironment in the first case. [79]. Furthermore, the lytic activity of neutrophils in vitro co-cultured with tumor cells that were engineered to express GM-CSF was 3–5 fold higher compared to parental tumor cells in which culture medium was supplemented with GM-CSF [79]. Therefore, the targeted delivery and subsequent release of cytokines at the site of the tumor may be of interest to increase local cytokine concentrations. Secondly, the presence of additional cytokines in the tumor microenvironment can obviously impact on the efficacy of the infused cytokine, as already exemplified above by the negative impact of TGF-β on G-CSF therapy. Hence, it is imperative to consider and carefully delineate cancer type-specific microenvironmental factors in potential cytokine/chemokine-based therapeutic approaches. 

## 3. FcR-Mediated Neutrophil Activation; Targeting FcγRIIa (CD32a) and FcαRI (CD89) for Optimal Responses

**Figure 2:**
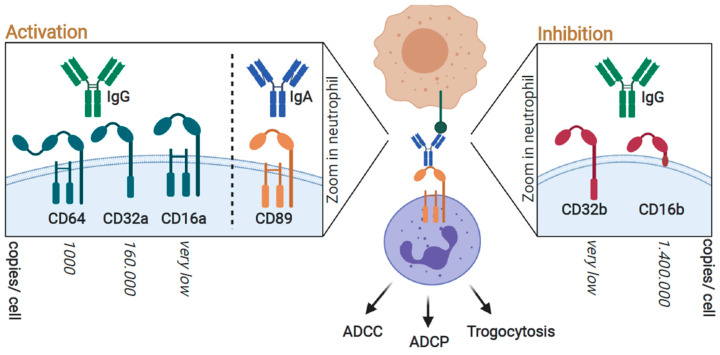
Antibody-mediated FcR activation of neutrophils. Neutrophils express various FcRs that are either activating or inhibitory, thereby respectively stimulating or repressing neutrophil-mediated antibody-dependent cellular phagocytosis (ADCP), antibody-dependent cellular cytotoxicity (ADCC), and trogocytosis. Among the activation receptors are the immunoglobulin G (IgG)-binding FcRs; CD64, CD32a and CD16a. In addition, neutrophils express the immunoglobulin A (IgA)-binding FcR CD89. Inhibitory IgG-binding FcRs that are expressed by neutrophils comprise CD32b and CD16b.

Perhaps the main way that neutrophils are involved in anticancer activity is by the activation of Fc-receptors (FcRs) upon binding by the immunoglobulin (Ig) domain of therapeutic antibodies. Most therapeutic antibodies currently in the clinic or in clincal trials are of the immunoglobulin G (IgG) isoform that can interact with activating Fcγ receptors (FcγRs) and trigger antibody-dependent cellular phagocytosis (ADCP), antibody-dependent cellular cytotoxicity (ADCC), and trogocytosis [31], [80,81,82]. Human neutrophils express the activating Fcy receptors, FcγRI (CD64), FcγRIIa (CD32a), FcγRIIc (CD32c), and FcγRIIIa (CD16a) [83,84,85,86], as well as the inhibitory FcγRIIb (CD32b) [87] and FcγRIIIb (CD16b) [83,84,85], [88] (Figure 2). Although CD64 is the high-affinity receptor and responsible for the anticancer activity of IgG antibodies on natural killer (NK) cells, IgG antibody-induced signaling on neutrophils is mainly dependent on CD32a [89], which is due to the fact that CD32a is the most abundant activating FcγR on neutrophils (160,000 copies/cell [90]. In contrast, neutrophils express 1000 copies of CD64 [90], although the expression of CD64 is upregulated by pro-inflammatory cytokines, such as G-CSF [91,92] and IFNγ [81,93]. In addition, neutrophils express low levels of CD16a that contribute to the antibody-mediated activation of neutrophils [84]. Notably, polymorphisms in CD32a (131-histidine(H)/arginine(R)) and CD16a (158 valine(V)/ phenylalanine(F)) may affect the efficacy of antibody-based cancer therapy [94]. Specifically, the homozygous histidine (H)/arginine (R) polymorphism at position 131 of CD32a (131 H/H) and CD16A (158 V/V) increased the response rate of rituximab [95] and trastuzumab [96] in respectively lymphoma and breast-cancer patients, whereas colorectal cancer patients with this specific polymorphism had a lower median progression-free survival time when treated with cetuximab [97]. Human IgG binds better to 131 H/H than 131 R/R, which may explain the improvement in efficacy for rituximab and trastuzumab [98]. Furthermore, a large controled study demonstrated that the H131R and V158F genotypes did not correlate with trastuzumab efficacy in HER2-positive breast cancer [99]. Differences in affinity for FcRs between antibodies may impact on the correlation between therapy efficacy and FcR polymorphisms. In addition, adjuvant treatment regimen, e.g., combination with chemotherapy, may influence the outcome of these studies.

**Figure 3:**
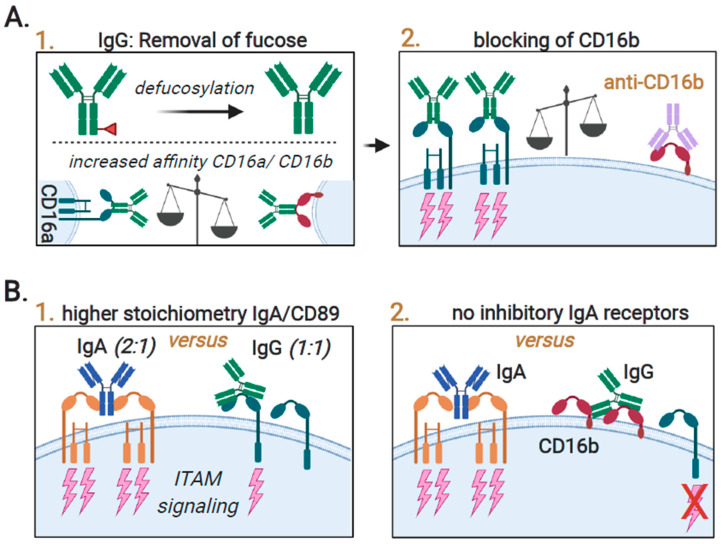
IgG versus IgA. (**A**) panel 1. The removal of fucose groups from antibodies (defucosylation/afucosylation) increases affinity for FcγRs and hence increases the activation of natural killer (NK) cells. However, neutrophils also express the inhibitory FcγR CD16b that is stronger bound by defucosylated antibodies as well, shifting the balance toward inhibitory signaling. (**A**) panel 2. CD16b-blocking antibodies prevent the interaction of therapeutic defucosylated antibodies to CD16b, shifting the balance toward activation. (**B**) panel 1. IgA binds with a higher affinity and stoichiometry to CD89 than IgG to CD32a. Specifically, IgA binds in a bivalent (2:1) conformation to CD89, resulting in the activation of four immunereceptor tyrosine-based activation motifs (ITAMs). In contrast, IgG binds in a monovalent (1:1) conformation to CD32a, yielding only one active ITAM motif. (**B**) panel 2. Whereas neutrophils express very high levels of CD16b, a non-signaling decoy inhibitory receptor for IgG, they do not express inhibitory FcRs for IgA.

Antibodies also interact with inhibitory FcγRs on neutrophils, with FcγRIIb (CD32b) (2000 copies per cell [90]) transmitting immunoreceptor tyrosine-based inhibitory motif (ITIM)-mediated inhibitory signaling [87,100,101]. Furthermore, neutrophils express the low-affinity FcγRIIIb (CD16b), which is a glycosylphosphatidylinositol (GPI)-anchored receptor lacking intracellular signaling motifs that likely has a decoy function [101,102]. Resting human neutrophils express CD16b at a very high level (1,400,000 copies/cell [90]), whereby IgG binding to resting neutrophils is predominantly mediated by CD16b [103,104]. In comparison, resting neutrophils only express 160,000 copies of CD32a [90]. In line with its inhibitory function, the blocking of CD16b using a F(ab’)2 of a CD16b antibody prominently increased both trogocytosis and ADCC induced by cetuximab and trastuzumab [88]. Of note, CD16b expression has also been shown to limit the efficacy of antibodies modified for increased affinity for the activating CD16a receptor. Specifically, the removal of fucosyl groups (i.e., defucosylation or afucosylation) increases antibody affinity for the activating receptor CD16 and is a strategy pursued to potentiate ADCC by NK cells [105,106,107]. However, defucosylation also increases the affinity of antibodies for CD16b by 7 to 15 fold [88,103,105,108]. Correspondingly, defucosylated trastuzumab had reduced neutrophil-mediated ADCC and trogocytosis activity compared to normal trastuzumab, which was reversed by blocking CD16b [88] (Figure 3A). Similarly, an afucosylated form of cetuximab, comprising point mutations S239D and I332E, increased the anticancer activity of NK cells but strongly reduced the ADCC of cancer cells by neutrophils from 60% to 10% [104]. The latter was not observed when using neutrophils that lacked CD16b expression (isolated from paroxysmal nocturnal hemoglobinuria patients) [104], again pointing toward the inhibitory function of CD16b that may be exacerbated upon antibody engineering. In contrast, both the afucosylated CD20 antibody obinutuzumab and defucosylated rituximab did efficiently activate neutrophils [103,109], with an enhanced induction of phagocytosis compared to the standard benchmark rituximab [103]. Of note, the clinical efficacy of obinutuzumab did not always outperform rituximab, while having an increased risk of toxicity [110,111]. The apparent discrepancy in effects on neutrophil activity by afucosylated/defucosylated antibodies may stem from the ability and/or preference of antibodies to bind either the activating CD32a or inhibitory CD16b receptor. Indeed, when a defucosylated derivative of cetuximab was modified to have enhanced affinity to CD32 as well, neutrophil anticancer activity was restored to that of the unmodified antibody [104]. Furthermore, it was suggested that binding to CD16b inhibits ADCC, but it may promote phagocytosis, as the GPI domain and the ectodomain are able to modulate the signaling of other receptors within the same lipid rafts [88]. Indeed, the blocking of CD16b using anti-CD16b F(ab’)2 fragments reduced the phagocytosis of cancer cells, bacteria, and zymosan particles [88,103,112]. Importantly, CD16b is uniquely expressed on granulocytes and not expressed by other types of immune cells [113,114] and may thus be an important consideration for the design of IgG-based neutrophil targeting in cancer immunotherapy. Notably, CD16b is not expressed in mice [113], and mouse studies may thus overestimate the therapeutic impact of CD16-targeting antibodies compared to the human situation.

Furthermore, the outcome of IgG-mediated neutrophil anticancer activity is influenced by local inflammatory conditions. For instance, the activation of neutrophils with G-CSF or IFN-γ upregulated CD64 and strongly reduced the expression of CD16b [88]. Correspondingly, the inhibition of CD16b using CD16 F(ab’)2 did not potentiate ADCC and trogocytosis by these activated neutrophils. Such upregulation of CD64 and downregulation of CD16 has also been observed on HDNs isolated from patients with multiple myeloma [115]. However, although CD64 is an activating FcR, these HDNs from myeloma patients were less effective in phagocytosis compared to healthy controls, which was caused by the increased expression of arginase-1, which is an enzyme that is immune inhibitory. Indeed, arginase-1 inhibitors reactivated the HDNs of myeloma patients [115]. Reversely, the immunosuppressive cytokine TGF-β may in the tumor microenvironment downregulate CD64 expression, as for instance reported for monocytes [116]. Thus, the balance of receptor expression as well as neutrophil polarization status in the tumor microenvironment is difficult to predict. An additional layer of complexity in this respect is the fact that CD16b can be proteolytically shed by ADAM17, which is a matrix metalloprotease often highly expressed in the cancer microenvironment [117]. Taken together, although the “net” activity of defucosylated/afucosylated antibodies may be improved compared to parental antibodies via the stronger activation of NK cells, this modification generally has a negative impact on the activity of neutrophils.

In addition to using IgG1 formats, the specific activation of neutrophils can also be achieved by the use of antibodies of the IgG2 isotype. Specifically, whereas panitumumab (anti-epidermal growth factor receptor (EGFR) IgG2) was inactive in triggering ADCC by mononuclear cells, it induced tumor cytotoxicity when using neutrophils as effector cells to a similar extend as zalutumumab (anti-EGFR IgG1) [50]. In contrast, panitumumab was also ineffective in inducing neutrophil-mediated tumor cell lysis in another study, which relied on the activation of inhibitory CD16 signaling [118]. Further, soluble IgG2 in contrast to membrane-bound IgG2 does not interact with the inhibitory receptor CD16b, whereas both soluble as well as membrane-bound IgG1 does [119].

Another important FcR that is a potential prominent, yet less explored, target for antibody-based therapeutics is FcαRI (CD89). CD89 is highly expressed on neutrophils, although it is expressed ≈ 2 times lower than CD32a [120], as well as on other myeloid cells [121]. CD89 is bound by immunoglobulin A (IgA), the second most prevalent immunoglobulin in the serum reviewed by [122]. In humans, IgA is comprised of the subclasses IgA1 and IgA2, of which IgA1 is about nine times more prevalent in serum compared to IgA2. IgA1 has a 13 amino acid insertion in the hinge region that contains five glycosylation sites [123], whereas IgA2 itself contains a higher number of N-glycosylation sites [124,125]. IgA2 was recently shown to have a higher affinity for CD89 on neutrophils compared to IgA1 due to reduced N-linked terminal sialic acids, with desialylation restoring IgA1 activity to that of IgA2 [126]. In line with these data, an IgA2 isoform of an EGFR antibody was superior over IgA1 in recruiting neutrophils [127]. Importantly, both IgG and IgA isoforms seem to bind equally to their target antigen, as has been shown for EGFR, with both variants blocking EGF binding and inhibiting EGFR phosphorylation [127]. Nevertheless, in a head-to-head comparison using a CD20 antibody of the IgG and IgA isotype, ADCC was more effectively induced by the IgA than the IgG antibody [120]. Furthermore, neutrophil-mediated tumor cell killing was stronger using IgA compared to IgG antibodies for various cancer antigens, e.g., epithelial cellular adhesion molecule (EpCAM) (colon carcinoma) [128], human epidermal growth factor receptor-2 (HER-2) (mammary carcinoma) [129], EGFR (epithelial, colorectal, and renal cell carcinoma) [130], CD30 (B- and T-cell lymphoma) [131], HLA class II [132], and CD20 (B-cell lymphoma) in in vitro studies [127] and in vivo studies [133,134]. 

In line with these studies highlighting the more prominent role of CD89, immature bone marrow-derived neutrophils mobilized with G-CSF efficiently eliminated tumor cells via CD89, but they did not trigger CD64-mediated anticancer responses [135]. Similarly, the use of an IgA isoform was also superior over IgG isoforms in recruiting monocytes/macrophages as well as neutrophils [130,133]. This superior activity of IgA has been attributed to the stronger affinity of CD89 for IgA compared to the affinity of IgG for CD32a, >10^7^ versus 10^6^ respectively [136,137]. Indeed, the binding of IgA to CD89 is more stable, and in contrast to other Fc receptors, which form 1:1 complexes with the Fc regions of their target antibodies, the interaction between CD89 and IgA1 results in the formation of a 2:1 complex [120,138]. This bivalent binding recruits four instead of one immunereceptor tyrosine-based activation motif (ITAM) domains, yielding stronger signaling (Figure 3B; panel 1). Furthermore, unlike IgG1-mediated FcγR signaling, the IgA-signaling pathway is not subject to the inhibition of signaling via CD16b [88] (Figure 3B; panel 2). However, it should be noted that the serum half-life of IgA antibodies is much shorter compared to IgGs [139], which may limit efficacy or necessitate more frequent infusions. These problems may be resolved by engineering IgA with lower levels of terminal galactosylation formulations with increased stability [140,141]. Furthermore, the in vivo evaluation of IgA antibodies is more challenging than IgG antibodies as mice possess a completely different IgA system and do not express an CD89 homologue, and hence, transgenic mouse models are required to study the efficacy of IgA antibodies [142]. 

**Figure 4:**
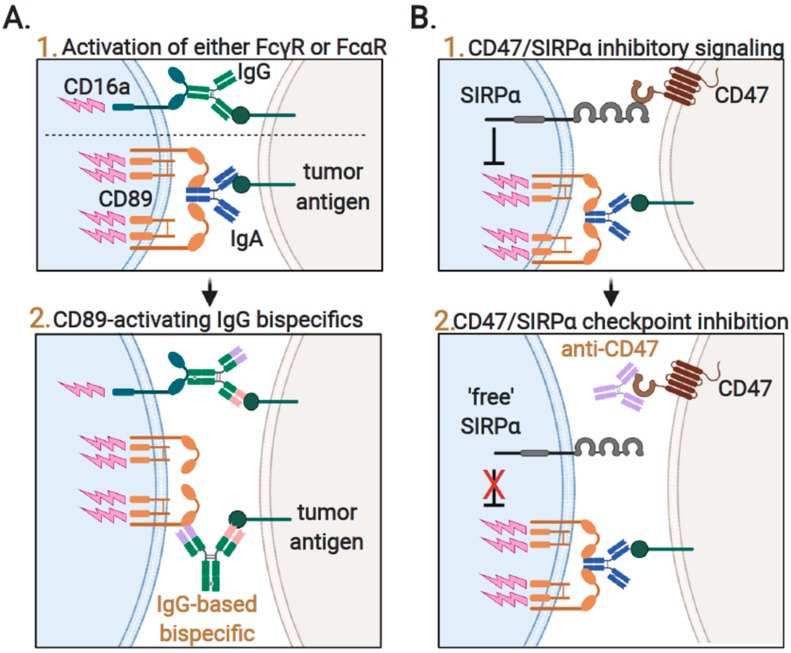
Antibody-based therapeutic strategies to improve neutrophil-mediated anticancer immune responses. (**A**) panel 1. Whereas IgA outperforms IgG in terms of neutrophil activation, IgGs are the optimal isotype to activate natural killer (NK) cells. Therefore, a combination of both isotypes would be needed to activate all types of immune cells. (**A**) panel 2. CD89-activating IgG-based bispecifics have been designed, which activate neutrophils as well as NK cells. Furthermore, the IgG backbone overcomes various problems of IgA antibodies, such as the reduced serum half-life of IgA. (**B**) panel 1. The interaction of cancer cell expressed CD47 with signal-regulatory protein alpha (SIRPα) expressed on neutrophils inhibits antibody antibody-dependent cellular phagocytosis (ADCP), antibody-dependent cellular cytotoxicity (ADCC), and trogocytosis, even in the presence of activating IgG/IgA antibodies. (**B**) panel 2. CD47 blocking antibodies have been designed to “release the break” on the activation of phagocytes.

In addition, it should be noted that IgG isoforms have the critical benefit of efficiently triggering and activating NK cell-mediated cytotoxicity, which argues for the simultaneous use of both IgA and IgG mAbs (Figure 4; panel 1). Indeed, a combination of IgA and IgG mAbs targeting EGFR and HER2 displayed increased cytotoxicity compared to either isoform alone both in vitro as well as in vivo [143]. In addition, the use of a trastuzumab [144] or an anti-CD20 [145] tandem IgG1/IgA2 format yielded superior activity over the parental IgG antibody by efficiently recruiting all types of FcR-expressing immune cells. In contrast, although anti-EpCAM IgA1 induced a higher tumor-specific cell lysis than IgG1 by purified PMNs, the combination of IgA1 and IgG1 reduced tumor cell death compared to IgA alone [146]. Furthermore, when using whole blood as effector cells, tumor cell lysis induced by IgG1 alone was stronger compared to the combination of IgA1 and IgG1. This discrepancy in efficay of IgA1 and IgG1 combinations was attributed to triggering of the inhibitory receptor CD32b by IgG1, and indeed CD32b F(ab’)2 fragments restored ADCC [146]. Furthermore, in case the intensity of IgA-mediated triggering of CD89 on neutrophils is too low, inhibitory ITAM signaling can prevail [147].

Alternatively, bispecific antibodies comprising a human IgG1 bispecific antibody with additional specificity for CD89 can be exploited (Figure 4; panel 2). For instance, an IgG1-based bispecific targeting CD20 and CD89 effectively recruited and activated CD89-positive neutrophils and macrophages to CD20-positive cancer cells, yielding prominent in vitro and in vivo anticancer activity [148,149]. Similarly, CD89-directed bispecifics targeting CD19, major histocompatibility complex (MHC) class II or CD30, induced the neutrophil-mediated lysis of malignant B cells [131,149,150]. In addition, by using such bispecific IgG formats, IgG1 stability and half-life can be exploited for the effective recruitment and activation of IgA FcRs.

In conclusion, neutrophils can be activated using IgG-based antibodies via pro-inflammatory FcγRs CD64, CD32a, and CD16a, although this type of antibody is subject to potential inhibition by inhibitory receptors such as CD32b and CD16b. The activation of neutrophils via the pro-inflammatory FcαR CD89 using IgA antibodies is more effective, but it is also associated with potential drawbacks such as poor half-life. Continued development in this field, evidenced by IgA/IgG chimeric formats or the bispecific IgG-based antibody formats referenced above, yield important steps forward toward fully exploiting the antibody-mediated activation of neutrophil anticancer activity. 

### 3.1. Neutrophil-Mediated Cytotoxic Activity toward Cancer Cells; Targeted Anticancer Activity by Antibody-Dependent Cellular Cytotoxicity (ADCC) 

In a therapeutic setting, antibody-mediated targeting of neutrophil activity to cancer antigens can activate ADCC, which will trigger the release of reactive oxygen species (ROS) during a so-called “oxidative burst”. In addition, the content of neutrophil granules are released, whereby cytotoxic molecules such as elastase, myeloperoxidase, cathepsins, and defensins (primary granules), lactoferrin, arginase, and matrix metalloprotease 9 (secondary and tertiary granules) can eliminate the targeted cancer cell. ADCC has been shown to contribute to neutrophil-mediated cytotoxicity for various therapeutic antibodies currently in clinical use. For instance, rituximab treatment induced neutrophil-mediated ADCC in B cell lymphoma in in vitro assays [151], whereas cetuximab induced neutrophil-mediated ADCC in head and neck cancer [118], skin squamous cell carcinoma [104], and colon carcinoma [152]. The ADCC activity of cetuximab correlated with the expression level of EGFR on the cell surface of the target cells [153]. Similar neutrophil-dependent ADCC has been reported for antibodies targeting HER2/Neu (trastuzumab) [82,88,89], CCR4 [154], EGFR (panitumumab and zalutumumab) [50], EpCAM [146], and CD52 (alemtuzumab) [155]. Indeed, the alemtuzumab-mediated depletion of lymphocytes was inhibited or even abrogated in the absence of neutrophils in murine lymphoma models [155,156,157]. A similar loss of ADCC activity upon treatment with rituximab was detected upon neutrophil depletion in a mouse model [158]. 

In addition, although not having a direct impact on activating neutrophils, an antibody-based activation of the complement system can stimulate neutrophil-mediated anticancer immune responses. In brief, the cleavage products C3a and C5a that are formed during the complement cascade are potent neutrophil chemoattractants, which increase tumor infiltration by neutrophils during antibody immunotherapy in mice [159,160,161,162]. This neutrophil recruitment via complement is further stimulated by treatment with β-glucans [159,161,162], which is a polysaccharide-based supplement that is also being tested in clinical trials for the treatment of cancer [NCT00857025] [NCT00682032] [NCT03461354]. Notably, high expression levels of the inhibitory membrane-bound complement regulatory proteins on the surface of cancer cells limit complement activation and subsequent neutrophil cytotoxicity, which can be prevented by the use of blocking antibodies [160,163]. Thus, the optimization of antibodies for potently activating the complement system, e.g., by engineering the Fc region to increase C1q binding affinity [164], may positively impact on neutrophil-mediated anticancer responses.

### 3.2. Neutrophil-Mediated Engulfment of Cancer Cells; Antibody-Dependent Cellular Phagocytosis (ADCP)

Neutrophils are profesional phagocytes with the capability to engulf and eliminate pathogens, cell debris, as well as cancer cells. Phagocytosis is a complex process that includes the recognition, internalization, transport, and eventual degradation of engulfed material. Of note, the intracellular processing of ingested material differs of neutrophils diverges from the typical endocytosis pathway such as that used by macrophages. Specifically, as neutrophils only have a small endosomal compartment, the content of the neutrophil phagosome is typically degraded by fusion of the phagosome with secretory vesicles and granules [as reviewed by [165]. 

For the purpose of cancer immunotherapy, the antibody-mediated activation of neutrophil phagocytosis, also called antibody-dependent cellular phagocytosis (ADCP), is of most importance. The neutrophil-mediated phagocytosis of cancer cells has been reported for CD20-targeting antibodies in ex vivo assays. For instance, the CD20 antibody rituximab induced the phagocytosis of B cell lymphoma cells by isolated human neutrophils in various studies [103,109,166,167,168], ranging from 38% to 60% after 24 h of incubation. Similarly, CD20-targeting antibodies obinutuzumab and ofatumumab yielded similar levels of neutrophil-mediated phagcocytosis [103,167]. As described above, neutrophil-mediated phagocytosis by these antibodies was enhanced by using a glycoengineered defucosylated form [103,109], which was attributed to differential binding to FcRs. Similar neutrophil-mediated ADCP has been reported for trastuzumab [166] and rituximab [167].

Of note, neutrophils isolated from the spleen of leukemic mice displayed reduced phagocytic capacity ex vivo compared to neutrophils from control animals due to reduced Toll-like receptor (TLR) expression levels, which was restored by stimulation with IL-15 and G-CSF [169]. Indeed, myeloid cells from human leukemia patients often also have downregulated TLR expression levels [170,171,172]. Interestingly, the treatment of cancer patients with G-CSF in the form of pegfilgrastim, a pegylated recobinant form of G-CSF, is already approved to prevent neutropenia and is well-tolerated even in combination with rituximab [173,174,175,176]. Although the overall response rate of the combination of rituximab with pegfilgrastim did not differ from treatment with rituximab alone, the authors mentioned a “remarkably long” duration of the remission phase and argue for a follow-up study where rituximab monotherapy is compared one on one with the combination with pegfilgrastim. As neutrophil counts are increased by pegfilgrastim, neutrophil-mediated ADCP may have a positive effect on rituximab therapy. Indeed, the pre-treatment of human neutrophils with pegfilgrastim in ex vivo assays significantly increased rituximab-induced ADCP, with a maximal increase from 15 to 40% ADCP [168]. In addition, in an in vivo model, the addition of pegfilgrastim to rituximab treatment strongly reduced tumor volume compared to single treatments, with all mice receiving pegfilgrastim having increased neutrophil counts in the spleen, blood, and tumor. Thus, the addition of pegfilgrastim to antibody therapy may not only prevent severe neutropenia, but it may also contribute to therapeutic efficacy by enhancing ADCP by neutrophils.

Taken together, although clear ADCP has been observed using isolated human neutrophils in ex vivo assays, it should be noted that the contribution of neutrophils to ADCP is generally not investigated in clinical trials, and as a result, clear clinical corroboration of the importance of ADCP is lacking. However, a strong inhibition of neutrophil ADCP due to complement activation and the presence of excess levels of competing IgGs was reported in whole blood assays in two publications [103,167], although in a similar third study, no difference was detected between ADCP by isolated neutrophils and in whole blood [109]. Thus, although neutrophil-mediated ADCP can clearly occur in a preclinical setting, studies to dilineate this effect in clinical settings are required. 

### 3.3. Trogocytosis; Lysing Cancer Cells by Biting off Parts of the Plasma Membrane

Although the neutrophil-mediated phagocytosis of cancer cells is well documented, cancer cells are typically larger than neutrophils (e.g., an average diameter of 12–22 µM for cancer cells vs. ≈8 µm diameter for human neutrophils, respectively [177,178]). Therefore, it is conceptually a challenge for a small neutrophil to engulf a complete cancer cell. Indeed, neutrophils were recently reported to not phagocytose B cell chronic lymphocytic leukemia (B-CLL) cells but rather to only perform trogocytosis [179], a process in which parts of the plasma membrane of the target cell are taken. At the same time, neutrophils did efficiently phagocytose small beads with a diameter of 4.5 µm [179]. Further, no phagocytic uptake of CD20 antibody-opsonized B-CLL cells was detected by purified neutrophils, as determined using live-cell time-lapse microscopy [179]. In line with these observations, the total cell count of B-CLL cells did not significantly decrease, despite a clear increase in the number of PMNs that stained positive for the dye used to stain the B-CLL cells [179]. Thus, B-CLL appears to not be phagocytosed by neutrophils, although earlier studies did confirm the capacity of neutrophils to phagocytose not only by flow cytometry but also by confocal microscopy or microscopic analysis of cytospin [103,109,167]. The sequential uptake of membrane parts by neutrophils can eventually trigger loss of membrane integrity and lead to cell lysis, which is a type of cell death called “trogoptosis” [82]. Trogocytosis by neutrophils has been demonstrated in vitro for various clinically used therapeutic antibodies, i.e., antibodies directed at CD22 (epratuzumab) [180], HER2 (trastuzumab) [82], EGFR (cetuximab) [82], and CD20 (rituximab and obinutuzumab) [179], with rituximab proving to be more efficient in inducing trogocytosis than obinituzumab. 

Importantly, neutrophils containing a piece of HER2-positive membrane were detected in biopsies of breast cancer patients using immunohistochemistry as well as flow cytometry [82]. This finding provides evidence for the potential clinical relevance of neutrophil-mediated trogocytosis. Furthermore, in a murine melanoma model, trogocytosis was also demonstrated using the CD47-blocking peptide SSL6, in which active trogocytosing neutrophils were detected using intravital microscopy [82]. Thus, trogocytosis seems to be one of the mechanisms for neutrophils to eliminate cancer cells that may be relevant for clinically used therapeutic antibodies. Of note, the efficiency of trogocytosis is, similar to phagocytosis, increased by neutrophil-activating stimuli, such as by innate checkpoint inhibition (described in Section 2D) [82]. 

However, trogocytosis does not always result in cell death, as CD20 antibodies triggered the trogocytosis of B-CLL cells without affecting cell viability [179]. In this respect, trastuzumab-opsonized breast cancer cells that died via trogocytosis lost a significantly larger percentage of their plasma membrane compared to cells that underwent trogocytosis yet survived the treatment [82]. Consequently, the amount of “bites” and the size of the eventual “hole” in the plasma membrane seems to determine whether a cell will lyse due to trogocytosis or not. Furthermore, antibody-mediated trogocytosis is also known to downregulate the surface expression of target antigens on cancer cells [82,179,180,181]. For instance, treatment with rituximab and obinutuzumab down-regulated the expression of CD20 in B-CLL cells due to neutrophil-mediated trogocytosis [179]. Furthermore, HER2/Neu expression was reduced upon neutrophil trogocytosis of breast cancer cells opsonized with tastuzumab [82]. Although these findings implicate trogocytosis in the escape of cancer cells from therapy due to a loss of target antigen, neutrophil-mediated downregulation of CD38 on lymphoma cells during daratumumab treatment was detected in both responders as well as non-responders [182]. Thus, it remains to be determined whether trogocytosis can contribute to resistance to antibody therapy due to a loss of antigen expression or not. 

For trogocytosis to occur, both adhesion molecule and FcγR-mediated interaction appears to be required, with both interactions facilitating the repeated close contacts between neutrophil and target cells characteristic for trogocytosis [82,179,183]. Indeed, the induction of trogocytosis in cancer cells is dependent on both CD18/CD11b and FcγR interactions, with CD18, CD11b, and FcR-blocking antibodies reducing trogocytosis [82]. Furthermore, the inhibition of downstream FcγR signaling pathways using syk inhibitors abolished cancer cell trogocytosis [82]. Similarly, using a pharmacological inhibitor library screen, trogocytosis was found to rely on the activity of phosphoinositol-3 kinase (PI3K), myosin light-chain kinase, and intracellular calcium flux. Of note, the inhibition of these signaling molecules also suppresses neutrophil-mediated ADCC [82], suggesting that trogocytosis could be a part of ADCC-mediated anticancer activity by neutrophils. Similar to neutrophil-mediated ADCC, trogocytosis is also inhibited by CD16b interaction of the opsonizing antibody, with treatment in the presence of blocking CD16 F(ab’)2 increasing the level of trogocytosis [88]. Cell death due to trogocytosis did not require the release of granules (containing granzymes and perforins), as the pharmacological inhibition of perforins did not affect cell death [183]. In line with these data, neutrophils from patients with a genetic mutation that abrogates the release of granules were not impaired in their ability to induce antibody-mediated trogocytosis [82,183]. Similarly, neutrophils incapable of producing reactive oxygen species were still able to induce trogocytosis, with a pharmacological inhibition of nicotinamide adenine dinucleotide phosphate (NADPH) oxidase not affecting cell death [183]. Thus, cell death due to trogocytosis is more likely a separate cytotoxic effector mechanism employed by neutrophils to eliminate cancer cells.

Taken together, neutrophils can kill cancer cells via trogocytosis, and this mechanism is likely to be predominant over phagocytosis when the target cell is too big for complete ingestion. The exact contribution of this process to anticancer activity of therapeutic antibodies remains to be identified, although it is likely that trogocytosis occurs in patients during antibody therapy.

### 3.4. Augmenting Neutrophil-Mediated ADCP and ADCC by Targeting of Innate Immune Checkpoints

In recent years, the targeting of checkpoints in T cell immunity has taken center stage. Similarly, neutrophil activity is kept in check by immunoregulatory checkpoints that can be exploited by cancer to silence neutrophil cytotoxicity. One of the best established in this respect is the overexpression of the “don’t eat me” signal CD47 in cancers. CD47 is a 50-kDa membrane glycoprotein that is expressed by virtually all cells in the human body, where it functions as “marker of self”. By binding to signal regulatory protein α (SIRPα), among others expressed by neutrophils, it inhibits phagocytic uptake or trogocytosis (Figure 4B; panel 1). Cancer cells hijack this inhibitory CD47–SIRPα pathway by overexpressing CD47, which is associated with poor prognosis in both solid [184,185] and hematological cancers [3,186]. Furthermore, significantly lower CD47 expression levels were detected on breast cancer cells of human patients that responded with a complete response after trastuzumab plus vinorelbine therapy [187]. Furthermore, in DLBCL patients treated with standard therapy (rituximab–CHOP; cyclophosphamide, doxorubicin, vincristine, and prednisone), the high mRNA expression of CD47 associated with poor surival in the aggressive non Germinal Center B cell subtype (non-GCB) [188]. Thus, the overexpression of CD47 by cancer cells prevents elimination by myeloid cells and reduces the efficacy of antibody immunotherapy. Therefore, blocking CD47 is a promising therapeutic approach to augment the neutrophil-mediated killing of cancer cells (Figure 4B; panel 2). Indeed, F(ab’)2 fragments of the CD47-blocking antibody B6H12 enhanced neutrophil-mediated ADCC by trastuzumab from 40% by trastuzumab alone to 80% upon combination treatment, whereas the CD47 F(ab’)2 fragment alone had no effect [187]. Similarly, the knock-down of CD47 also potentiated neutrophil-mediated ADCC in these studies. Correspondingly, SIRPα blocking agents also increased neutrophil-mediated anticancer effects, with antibody KWAR23 promoting the neutrophil-mediated phagocytic removal of lymphoma cells by rituximab and breast cancer cells by trastuzumab [166]. In addition, in vivo, the combination of KWAR23 and rituximab or KWAR23 and vorsetuzumab (anti-CD70) reduced tumor growth and led to partial or complete remission in 67% of the animals. Here, the depletion of either neutrophils or macrophages increased tumor growth, highlighting the relevance of both cell populations [166]. Similarly, mice treated with both rituximab and an anti-mouse SIRPα antibody reduced lymphoma growth in a xenograft model, resulting in prolonged survival [189]. In addition, a humanized form of this antibody increased neutrophil-mediated trogocytosis as induced by rituximab, increasing from 30% with rituximab only to 60% in combination with SIRPα mAb, although SIRPα-only control was lacking [189]. In addition to antibodies, the SIRPα/CD47 axis can be blocked by the use of recombinant SIRPα protein, whereby the exogenously added SIRPα protein interacts with endogenous CD47 on cancer cells, thereby preventing interaction with endogenous SIRPα expressed on phagocytes. Indeed, the dual signaling protein SIRPα-4-1BB blocked the interaction of SIRPα with CD47 and induced in vitro neutrophil- and macrophage-mediated phagocytosis of cancer cells [190]. 

Of note, different SIRPα variants are expressed among the human population, with SIRPα1 and SIRPαBIT being the most frequent allele among European, Admixed American, and African populations [89,186,188]. Although there were no differences detected in the ADCC capacity of neutrophils between the different SIRPα genotypes [89], the anticancer efficacy of SIRPα blocking therapy is absent when targeting the wrong SIRPα variant, as an antibody specific for SIRPα1 only promoted trastuzumab-induced ADCC when using neutrophils from α1/α1-homozygous donors [187]. Hence, pan-SIRPα antibodies that recognize all SIRPα variants have been developed, with similar increased neutrophil-mediated anticancer effects in the presence of therapeutic antibodies [189]. Thus, blocking the CD47-SIRPα axis can increase the therapeutic effect of antibody immunotherapy at least partly by increasing neutrophil activity. Of interest, blocking of the CD47–SIRPα with an IgA antibody also potentiated neutrophil-mediated ADCC and trogocytosis in vitro and inhibited tumor growth [191], with the IgA-based antibody being more potent in neutrophil activation than an IgG-based antibody.

However, CD47 expression on neutrophils itself is required for neutrophil transmigration. Indeed, both CD47 and SIRPα targeting blocking antibodies inhibit fMLP, IL-8, or TNFα-induced migration of neutrophils through collagen-coated filters and epithelial cell layers [192,193,194]. This argues for a more specific targeting of CD47 at the site of the cancer cell, thereby possibly preventing inhibitory effects on neutrophils’ transmigration. In this respect, the bispecific tandem single-chain variable fragment (scFv) RTX-CD47 induced the phagocytosis of CD20-expressing, but not CD20-negative B cell lymphoma cell lines by neutrophils [195]. This therapeutic activity required simultaneous binding to CD20 and CD47 and was not detected in single CD47-positive cells [195]. Several other IgG-based CD47-targeting bispecific antibodies have been developed, among others CD20-CD47 [196] and CD70-KWAR23 [166] CD47-CD19 [197,198], CD47-MSLN [197], CD47-PDL1 [199,200], and PD-L1-SIRPα [201]. However, the impact of these bispecific antibodies on neutrophil-mediated phagocytosis and trogocytosis has not been delineated yet. Of note, since the RTX-CD47 bispecific antibody format lacks an Fc domain, the induction of phagocytosis was solely due to the inhibition of the CD47–SIRPα axis. Indeed, whereas initially the presence of an intact Fc domain was reported to be needed for CD47 antibody-mediated phagocytosis [186,187], we and others clearly demonstrated that CD47 blocking did not require the presence of an Fc domain [186,195,202]. Indeed, equal levels of phagocytosis were detected when using a F(ab′)_2_ fragments of CD47 or SIRPα blocking antibodies compared to Fc-containing antibodies [186,195]. Notably, CD47-mediated phagocytosis also did not depend on cancer cell expression of the pro-phagocytic molecule SLAMF7 [202], as reported before in an earlier paper [202].

In addition to CD47/SIRPα, other immunomodulatory proteins may be of interest to target for improved neutrophil activity. An interesting example hereof is siglec-9, which is a member of the sialic acid-binding immunoglobulin-type lectins (siglecs) that is prominently expressed on neutrophils. Binding to siglec-9 induces inhibitory signaling in neutrophils and prevents the “uptake of self” by for instance erythrocytes [203]. Cancer cells highjack this inhibitory pathway by overexpressing siglec-9 ligands and/or via hyper-sialylation of siglec-9 ligands [204]. Indeed, antibody-mediated blocking of siglec-9 activated neutrophils and increased tumor cell killing [204]. Of note, mucin-1, a glycoprotein often overexpressed in cancer, is a known binding partner of siglec-9, and this interaction leads to cancer cell growth [205]. Hence, siglec-9-blocking strategies may work as a “double edged sword” by releasing the brake on neutrophil activation and inhibiting mucin-1-mediated cancer cell growth. Interestingly, siglec-9 also inhibits T cell activity [206,207] and may, therefore, potentiate both innate and adaptive anticancer immune responses. 

In conclusion, the targeting of innate immune checkpoints represents a promising approach to increase the uptake of cancer cells by phagocytes and augment cancer immunotherapy with therapeutic antibodies. Indeed, CD47 and SIRPα blocking strategies are being studied in many ongoing trials and have demonstrated promising therapeutic effects in various malignancies, including DLBCL [5,208], AML, [3] and Sezary syndrome [209], with acceptable safety and toxicity profiles [NCT02216409] [NCT02953782] [NCT02678338] [NCT02953509] [NCT03248479] [NCT03013218] [NCT02663518]. Furthermore, additional targets such as siglec-9 are being explored and moving toward clinical evaluations. However, to date, the majority of those studies are focused on macrophages-mediated effects. Nevertheless, preclinical data highlight the contribution of neutrophils to the anticancer activity of checkpoint targeting with confirmation of this activity in clinical settings being awaited.

## 4. Neutrophil-Mediated Induction of Adaptive Anticancer Immune Responses

Neutrophils were initially classified as “simple” innate immune cells important for the immediate elimination of pathogens. However, evidence has emerged that neutrophils can also present antigens to T cells in the context of MHC. Specifically, neutrophils in early-stage lung cancer patients had characteristics of antigen-presenting cells and were capable of cross-presenting tumor antigens to T cells, leading to the development of anticancer T cell responses [210]. In addition, upon phagocytosing B cell lymphoma cells treated with anti-CD20 antibodies, the expression of MHC class II on neutrophils increased [109], which may facilitate antigen presentation to helper T cells. Although these are to our knowledge the only studies of antigen presentation by neutrophils in cancer to date, antigen presentation by neutrophils and the concomitant mounting of T cell immunity has been delineated in various non-cancer related studies as well [211,212,213,214,215]. In addition, neutrophils can modulate the activity of other antigen-presenting cell (APCs) such as DCs as initially demonstrated in the context of infections and Crohn’s disease [216,217,218,219,220]. In the context of cancer, the intra-tumoral injection of CpG oligonucleotides-B (CpG-B) inhibited tumor growth and triggered an initial strong influx of activated neutrophils into the tumor, followed by DC activation/maturation and induction of T cell-mediated anticancer immunity [221]. Importantly, the depletion of neutrophils hampered the tumoricidal effect of CpG-B treatment, leading to loss in DC activation/maturation and a reduced number of CD8^+^ T cells in tumor tissue and tumor-draining lymph nodes. 

Currently, most evidence on the role of neutrophils in T cell immunity has been obtained in the context of microbial infections. In brief, the early recruitment of neutrophils to the site of infection, especially in case of the airways, aids the efficacy of subsequent T cell responses [222,223,224]. In addition, CD8^+^ T cell responses are sustained by neutrophil help [225]. Similar neutrophil-dependent T cell activation has been described in recent studies in cancer. For instance, during tumor-take experiments in mice (in a G-CSF secretion colon cancer model), tumors were first infiltrated by neutrophils, followed by macrophages and T cells, respectively. The depletion of neutrophils strongly reduced CD8^+^ T cell infiltration and associated with tumor progression [226]. Similarly, the inoculation of G-CSF or GM-CSF secreting cancer cells in mice activated neutrophils, which was followed by the induction of T cell responses [47,49]. Furthermore, neutrophil depletion prevented the induction of T cell responses in melanoma-bearing mice, again abrogating antitumor immunity and leading to tumor progression [23]. In line with these data, the co-culture of CD8^+^ T cells isolated from autologous peripheral blood or tumor specimens with TANs isolated from colon carcinoma [227] augmented T cell proliferation, activation, and IFN-γ secretion compared to isolated T cell cultures. Similarly, TANs isolated from early-stage lung cancer patients induced the proliferation of T cells isolated from healthy donors of both CD4^+^ and CD8^+^ subsets [8]. Furthermore, in co-cultures with neutrophils, CD3/CD28-activated T cells differentiated more into a “central memory” phenotype, increasing from 19% in CD8^+^ monocultures to 45% in CD8^+^ co-cultured with neutrophils, which is a phenotype essential for anticancer immune responses [228]. In contrast to the above, mature neutrophils of multiple myeloma patients significantly decreased T cell proliferation upon triggering the CD3 receptor using a bispecific antibody, which was not observed when using neutrophils from healthy donors [229]. However, only the level of mature neutrophils, which had increased TGF-β signaling suggesting being N2 neutrophils, correlated with prognosis in these patients. 

In addition, the interaction between neutrophils and T cells increased the level of co-stimulatory molecules (i.e., 4-1BBL, OX40L, CD54, CD86) on the neutrophil surface, whereby T cell proliferation and activation was stimulated [8]. Indeed, the presence of blocking antibodies against these upregulated costimulatory molecules partly (CD54, CD86) or completely (OX40L, 4-1BBL) inhibited the stimulatory effect of TANs on T cell responses. In addition, TANs strongly suppressed protumoral IL-17 secreting γδ T cells in a murine model of melanoma and hepatocellular carcinoma [230]. Interestingly, in colon carcinoma patient samples, neutrophils frequently colocalized with CD8^+^ T cells in tumor regions. This combined tumor infiltration associated with a better prognosis than infiltration by CD8^+^ T cells alone [227], thus providing initial proof for the clinical relevance of neutrophil/CD8^+^ T cell interactions in cancer.

In addition, neutrophils release various pro-inflammatory mediators, e.g., cytokines and granule contents, that may impact on the development of adaptive anticancer immunity [as reviewed by [231]. For instance, the release of human neutrophil peptides, lactoferrin, α-defensins, and LL-37 are generally reported as having an activating effect on T cell immunity, whereby lactoferrin promotes the recruitment and activation of APCs [232] and α-defensins dose-dependently attract monocytes [233] and promote DC and T cell infiltration [234]. Furthermore, neutrophil-derived IFN-γ may orchestrate the cross talk with T cells in antitumor response [235]. In contrast, myeloperoxidase (MPO), elastase, and arginase mainly have a suppressive impact on T cell immunity, whereby elastase sheds IL-2 and IL-6 receptors on T cells [236], and arginase can result in the downregulation of TCRζ [237]. In addition, neutrophils can produce so-called “neutrophil exracellular traps” (NETs), which are extracellular neutrophil-derived structures composed of DNA decorated with antimicrobial peptides derived from neutrophil granules. These NETs are used by neutrophils to trap and subsequently kill pathogens and are typically attributed a pro-tumorigenic role in cancer. In contrast, NETs are also able to directly prime T cells, whereby the responsiveness of T cells toward their antigens is increased [238]. Furthermore, NET-producing neutrophils infiltrated into tumors of head and neck squamous cell carcinoma patients and were associated with better survival [239]. Thus, NETs may form a platform for T cell priming, and in spite of the typical protumoral role, they may in specific cases contribute to anticancer immune responses. 

Taken together, neutrophils can function as antigen-presenting cells themselves or stimulate other APCs to activate T cells. Furthermore, they can directly stimulate T cells by either cell/cell contact or secreted factors. Therefore, neutrophils seem to be more than “simple” innate immune cells, being involved in the initiation of efficient adaptive anticancer immune responses. 

## 5. Conclusions and Perspectives

As apparent from this review, neutrophils have prominent anticancer activity that can be exploited for cancer immunotherapy. Unfortunately, to date, the clinical evidence for the relevance of neutrophils in the fight against cancer remains limited due, on the hand, to the fact that most studies focus on the effects of macrophages and T cells and do not investigate the contribution of neutrophils. On the other hand, current treatment strategies are also not designed with neutrophils in mind, with the extensive focus on IgG isotype antibodies as the prominent example. Indeed, the full potential and therapeutic relevance of neutrophils will become only apparent as neutrophil-tailored drugs, such as IgA based therapeutic mono- and bispecific antibodies, enter clinical practice. In this respect, it has become apparent from the field of virology that neutrophils are critical players in the development of both humoral and T cell-mediated immunity against viral infections during antibody treatment and vaccination [240]. In line with this, a neutrophil-mediated induction of anticancer T cell immunity is also increasingly recognized as detailed in this review. Thus, with increasing knowledge taking the neutrophil into account for cancer immunotherapy becomes ever more important for the effective induction of innate and adaptive anticancer immunity.

Importantly, neutrophils found in the established tumor microenvironment are often attributed with immune inhibitory effects, which could be targeted to revert neutrophil activity. Interesting in this respect is the finding that the adaptive immune checkpoint programmed death-ligand 1 (PD-L1) also has a regulatory effect on TANs, with PD-L1 on TANs inhibiting T and NK cell responses [241,242]. In line with this finding, the PD1–PD-L1-axis also directly blocks neutrophil cytotoxicity, which is an effect that was reversed by the blocking of neutrophil PD-L1 [243]. Thus, neutrophils may be involved in the (re)induction of T cell-mediated immunity upon PD-1 checkpoint therapy. Interestingly, TANs were also shown to secrete high levels of arginase, which is an enzyme that cleaves the semi-essential amino acid L-arginine that is critical for lymphocyte proliferation and function [244,245]. High arginase levels in the tumor tissue and serum of cancer patients associate with dampened T cell-mediated immune responses and correlate with disease progression [245,246,247]. Furthermore, tumor cells secrete the N2-promoting cytokine TGF-β [65,248] or stimulate other cells to produce TGF-β [249], and mesenchymal stromal cells can inhibit neutrophil effector functions [250] and transform neutrophils into a T-cell-suppressive phenotype [251]. Any of these neutrophil-inhibitory features may be a target for immunomodulatory strategies to convert the TANs back into “tumor killers” that drive antitumor innate and adaptive immunity.

Notably, in the design of such neutrophil-based immunotherapy, it is imperative to consider the often detrimental effects of standard cytotoxic therapy on neutrophils, frequently leading to neutropenia in cancer patients undergoing treatment. Therefore, the dosing and timing of cytotoxic and neutrophil-targeted therapeutic strategies is pivotal to ensure an optimal window of therapy. In this respect, the infusion of ex vivo expanded neutrophils to overcome neutropenia, as has e.g., been explored in early clinical trials using neutrophils expanded from CD34^+^ hematopoietic stem cells [252,253], may well be combined with immunomodulatory strategies that ensure an anticancer polarization of neutrophils, such as CRISPR/Cas9 gene editing of N2-polarizing transcription factors. Such gene editing of hematopoietic stem cells can be achieved with high efficiency [254], with proof-of-concept for the effective modification of neutrophil activity recently generated for severe congenital neutropenia using *ELANE* knock-out [255] that in phagocytic functions, ROS production, and chemotaxis was similar to healthy donors.

Another emerging approach of interest is the use of neutrophils as carriers for drug-containing nanoparticles, since neutrophils are uniquely able to penetrate into the tumor microenvironment. For instance, neutrophils ex vivo loaded with liposomes containing paclitaxel suppressed postoperative glioma recurrence and increased survival in mice [256]. In addition, the injection of nanoparticles 24 h after antibody TA99 (specific for gp75) administration increased the neutrophil and nanoparticle accumulation in the tumor, whereby TA99 guided neutrophils into the tumor via ADCC. This treatment resulted in increased survival rates and has the advantage that it does not require ex vivo particle loading [257]. Further, neutrophils can be recruited to the tumor site by inducing an inflammatory response using a photosensitizer. Indeed, the combination of photosensitizer and CD11b-targeting nanoparticles carrying a photothermal therapeutic resulted in the elimination of tumor cells and prolonged survival of lung cancer bearing mice [258]. In a similar approach, neutrophil membranes can be used to deliver nanoparticles to the tumor [259]. Of note, in all of these studies, the loading of nanoparticles did not negatively impact on neutrophil functionality. Thus, such nanoparticle-based strategies may even be combined with ex vivo expanded neutrophils to equip such neutrophils with additional anticancer activity. 

In conclusion, with the increasing understanding of the contribution of neutrophils to anticancer immune responses, strategies tailored to more efficiently exploit neutrophil-mediated responses are being developed toward clinical application and are anticipated to translate into the development of effective innate and adaptive anticancer immunity.
